# Mental Health Issues in Massachusetts, New Hampshire, and Rhode Island: Findings From the Healthy Aging Data Reports

**DOI:** 10.1093/geroni/igaa057.312

**Published:** 2020-12-16

**Authors:** Taylor Jansen, Richard Chunga, Chae Man Lee, Shuangshuang Wang, Haowei Wang, Frank Porell, Nina Silverstein, Beth Dugan

**Affiliations:** 1 University of Massachusetts Boston, Boston, Massachusetts, United States; 2 University of Massachusetts Boston, Boston, United States; 3 Shandong University, Jinan, Shandong, China; 4 University of Massachusetts Boston, Needham, Massachusetts, United States

## Abstract

Mental health issues in older adults are prevalent, yet often undetected or untreated and can contribute to poor physical health, increased disability, and higher mortality rates. The current study describes state and local community rates of mental health indicators of older adults 65+ in MA, NH, and RI. Data sources used to calculate rates were: the American Community Survey (2009-2013 RI, 2012-2016 MA and NH), the Medicare Current Beneficiary Summary File (2012-2013 RI, 2015 MA and NH), and the Behavioral Risk Factor Surveillance System (2012-2014 RI, 2013-2015 MA, and 2014-2016 NH). Small area estimation techniques were used to calculate age-sex adjusted community rates for more than 150 health indicators. This research examines disparities in rates for 3 mental health indicators depression, self-reported poor mental health, and self-reported poor/fair health status. Depression rates: MA 31.5% (19.91-48.82%), RI 30% (19.7-38.5%), and NH 28.8% (18.26-40.56%). Self-reported poor mental health: RI 7.5% (4.8-12.5%), MA 7.0% (2.10-16.59%), and NH 6.9% (3.42-10.13%). Self-reported fair/poor health: RI 20.4% (8.6-38.8%), MA 18.0%, (7.2-34.38%), and NH 16.5% (13.31-21.60%). Results showed variability in rates across states. MA had the highest rates of depression, the greatest differences in rates, and access to the most mental health providers. RI had the highest community rates for poor physical and mental health, and the highest percentage of residents age 85+. Understanding the distribution of community rates makes disparities evident, and may help practitioners and policymakers to allocate resources to areas of highest need. Research funded by the Tufts Health Plan Foundation.

